# Long‐term B cell depletion associates with regeneration of kidney function

**DOI:** 10.1002/iid3.499

**Published:** 2021-07-29

**Authors:** Susanne V. Fleig, Franz F. Konen, Christoph Schröder, Jessica Schmitz, Stefan Gingele, Jan H. Bräsen, Svjetlana Lovric, Bernhard M. W. Schmidt, Hermann Haller, Thomas Skripuletz, Sibylle von Vietinghoff

**Affiliations:** ^1^ Department of Internal Medicine Division of Nephrology and Hypertension, Hannover Medical School Hannover; ^2^ Nephrology Section, Medical Clinic 1 University Hospital Bonn, Rheinische Friedrich‐Wilhelms University Bonn Germany; ^3^ Department of Neurology, Hannover Medical School Hannover; ^4^ Interdisciplinary Day Clinic, Hannover Medical School Hannover; ^5^ Nephropathology unit, Institute for Pathology Hannover Medical School Hannover

**Keywords:** cancer, cell migration, liver/hepatocytes

## Abstract

**Background:**

Chronic kidney disease (CKD) is a common condition that increases mortality and the risk of cardiovascular and other morbidities regardless of underlying renal condition. Chronic inflammation promotes renal fibrosis. Recently, renal B cell infiltrates were described in chronic kidney disease of various etiologies beyond autoimmunity.

**Methods:**

We here investigated B cells and indicators of tertiary lymphoid structure formation in human renal biopsies. Renal function was studied during long‐term B cell depletion in human patients with membranous nephropathy and with CKD of unknown origin.

**Results:**

Cytokine profiles of tertiary lymphoid structure formation were detected in human renal interstitium in a range of kidney diseases. Complex B cell structures consistent with tertiary lymphoid organ formation were evident in human membranous nephropathy. Here, B cell density did not significantly associate with proteinuria severity, but with worse excretory renal function. Proteinuria responses mostly occurred within the first 6 months of B cell depletion. In contrast, recovery of excretory kidney function was observed only after 18 months of continuous therapy, consistent with a structural process. Renal tertiary lymphatic structures were also detected in the absence of autoimmune kidney disease. To start to address whether B cell depletion may affect CKD in a broader population, we assessed kidney function in neurologic patients with CKD of unknown origin. In this cohort, eGFR significantly increased within 24 months of B cell depletion.

**Conclusion:**

Long‐term B cell depletion associated with significant improvement of excretory kidney function in human CKD. Kinetics and mechanisms of renal B cell aggregation should be investigated further to stratify the impact of B cells and their aggregates as therapeutic targets.

## Graphical abstract

B cell aggregates are recognized in a growing number of kidney diseases of diverse etiologies. We tested the effect of long‐term B cell depletion on renal function. Our data show long‐term improvement in two independent patient cohorts. This is consistent with the hypothesis that B cell‐directed intervention may be successful in chronic renal inflammation of diverse etiologies.

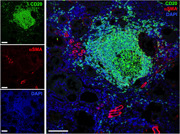

## 1 INTRODUCTION

B cells can directly infiltrate nonlymphoid organs including the kidney. Persisting inflammation can induce organized structures, which contain T cells and lymphatic vessels and conform to the definition of tertiary lymphoid structures (TLS).^1^, ^2^ TLS resemble lymph nodes in cell composition and architecture, but are confined by the host organ and displace the organ parenchyma. B cells are recruited by local stroma‐expressed chemokine CXCL13 (alternate name: B cell attractant 1).^1^, ^2^, ^3^ They in turn secrete lymphotoxins including lymphotoxin B (LTB),^4^ which promote perivascular stroma differentiation towards lymphoid tissue fibroblastic reticular cells and follicular dendritic cells,^5^, ^6^ thereby consolidating the new lymphoid structure.^7^

In the kidney, TLS were reported in a range of autoimmune diseases including ANCA‐associated glomerulonephritis, renal systemic lupus erythematodes, membranous glomerulopathy, and IgA nephritis.^8^, ^9^, ^10^, ^11^, ^12^ Complex B cell infiltrates also occurred in alloimmunity, namely kidney transplant rejection, both in humans and murine models.^13^, ^14^, ^15^, ^16^ More recently, renal TLS were described in the absence of an alloimmune stimulus, for example during long‐term follow‐up of murine ischemia‐reperfusion injury^1^, ^16^ and in severe human pyelonephritis.^17^

Therapeutic B cell depletion is clinically successful in a range of antibody‐mediated kidney diseases including renal allograft rejection and glomerulonephritides. For example, in membranous nephropathy, a common cause of the nephrotic syndrome, B cell depletion by rituximab achieves similar remission rates as previous protocols such as the Ponticelli regimen and calcineurin inhibitors.^18^, ^19^, ^20^ Current evidence includes several controlled trials^21^, ^22^ as well as a range of case series. Currently, depletion of auto‐ or alloantibodies and thereby abrogation of downstream innate immune activation and tissue destruction is considered to be the major mechanism of action. B cell depletion by rituximab also dissolved diffuse B cell infiltrates in the human kidney allografts,^14^ while short‐term B cell depletion was apparently ineffective for TLS dissolution, albeit in very limited patient numbers.^23^ In contrast, TLS disappeared in a murine SLE model after long‐term B cell depleting therapy.^24^

It is conceivable that diffuse B cell infiltrates and TLS may impair organ function even in the absence of antibody‐mediated immunity. If renal TLS form in chronic kidney disease (CKD) of various etiologies, long‐term B cell depletion should benefit also patients without a diagnosis of antibody‐mediated renal disease. We here studied B cell infiltration and markers of TLS formation in human glomerulonephritis and assessed the development of kidney function during long‐term B cell depletion in membranous glomerulonephritis and CKD of unknown origin.

## 2 MATERIAL AND METHODS

### 2.1 Study populations

Data analyses were carried out after ethics board approval (MHH 2018‐8172 and 2019‐8275) in accordance with the Helsinki Declaration. In the department of nephrology, patients with a histological diagnosis of membranous glomerulopathy were identified from the electronic records using the inpatient billing codes “membranous glomerulonephritis” and “nephrotic syndrome due to membranous nephropathy” 2015–2019 and from the records of specialist nephrology outpatient clinics. The lack of longer follow‐up among responders was due to recent start of therapy (*n *= 6), loss of follow‐up (*n *= 3), lack of follow‐up data for month 18 (with good response at month 24, *n *= 1), and start of hemodialysis (*n* = 1). At least one B cell measurement was performed in every patient after B cell depletion and these were negative. The records of the department of neurology at Hannover Medical School were screened for patients treated with B cell depletion with rituximab since 2012 for 24 months or longer. Patients with renal biopsy findings indicative of vasculitis were excluded (*n* = 2). Of 65 identified patients, 13 had an estimated glomerular filtration rate (eGFR) of 70 ml/min/1.73 m^2^ or less at start of therapy and were included into this analysis.

Laboratory values obtained at Hannover Medical School were recorded before start of rituximab and at the indicated intervals thereafter. eGFR was calculated using the Chronic Kidney Disease Epidemiology Collaboration (CKD‐EPI) formula,^25^ proteinuria was measured by dipstick analysis in the neurology cohort and as protein/creatinine ratio in spot urine samples in the nephrological patients.

### 2.2 Gene array data analysis

Tubulointerstitial LTB and CXCL13 expression were analyzed in public data sets from the European cDNA bank cohort, the Nephrotic Syndrome Study Network, and the Vasculitis Clinical Research Consortium^26^ obtained at NCBI (GSE104948 and GSE104954).

### 2.3 Immunostaining, confocal analysis, and quantification of B cell infiltrates in membranous glomerulopathy

In all available biopsies and a nephrectomy specimen from a patient with oxalosis, immunostaining with anti‐CD20 (1:50; clone L26; Agilent) of 3 µm dewaxed and rehydrated paraffin‐embedded kidney biopsy sections was conducted for 90 min after heat‐induced epitope retrieval with T‐ethylene diamine tetra‐acetic acid buffer (pH 9.0, Zytomed Systems), endogenous peroxidase activity (3% H_2_O_2_), and protein blocking (Zytomed). Negative controls omitting primary antibodies were included in all staining protocols. For detection, ZytoChem Plus horseradish peroxidase polymer system (mouse/rabbit; Zytomed) was used before 3,3ʹ‐diaminobenzidine chromogen (Zytomed) and counterstain by hemalum. Brightfield slides stained for CD20 were scanned at 40× original magnification (Aperio CS2, Leica Microsystems). Immunostained area was quantified in a pixel‐based approach with an interactively set threshold using an open‐source image analysis platform (QuPath, version 0.1.2, https://qupath.github.io/).^27^ The same threshold was used for all sections. Each section was controlled for staining quality, threshold, and annotation correctness by two pathologists blinded for staining, diagnosis, and case. Artifacts (e.g., tissue folds, chromogen irregularities) were excluded from the analysis. Groups were divided by B cell densities above and below median at start of therapy.

Selected samples were stained for confocal imaging using secondary goat antimouse‐AF488 (Invitrogen) and Cy3‐conjugated anti‐αSMA (1A3, Sigma). 4,6‐diamidino‐2‐phenylindole (DAPI, Invitrogen) nuclear counterstain was applied and slides were mounted in Immunoselect antifading mounting media (Dianova). Images were obtained with a Leica TCS SP8 confocal laser microscope and 20× multi‐immersion objective (Leica). Analysis was conducted with NIH ImageJ and GIMP (version 2.8).

### 2.4 Statistics

Statistical analysis was performed using Prism 8. Data are expressed as mean ± *SEM*. Test for Gaussian distribution was performed using D'Agostino and Pearson test. Two variables were compared using *t*‐tests or Kruskal–Wallis test for non‐normally distributed samples. Multiple comparisons were performed using one‐way ANOVA with Dunn's or Dunnett's multiple comparison tests as indicated. Multivariable regression was performed using SAS 9.4 (SAS Institute). *p* values less than .05 were considered significant, *p* values are indicated as follows: *p < .05, **p < .01, ***p < .001.

## 3 RESULTS

### 3.1 B cell infiltrates and mediators of lymphoid tissue organization in human kidney disease

**Figure 1:**
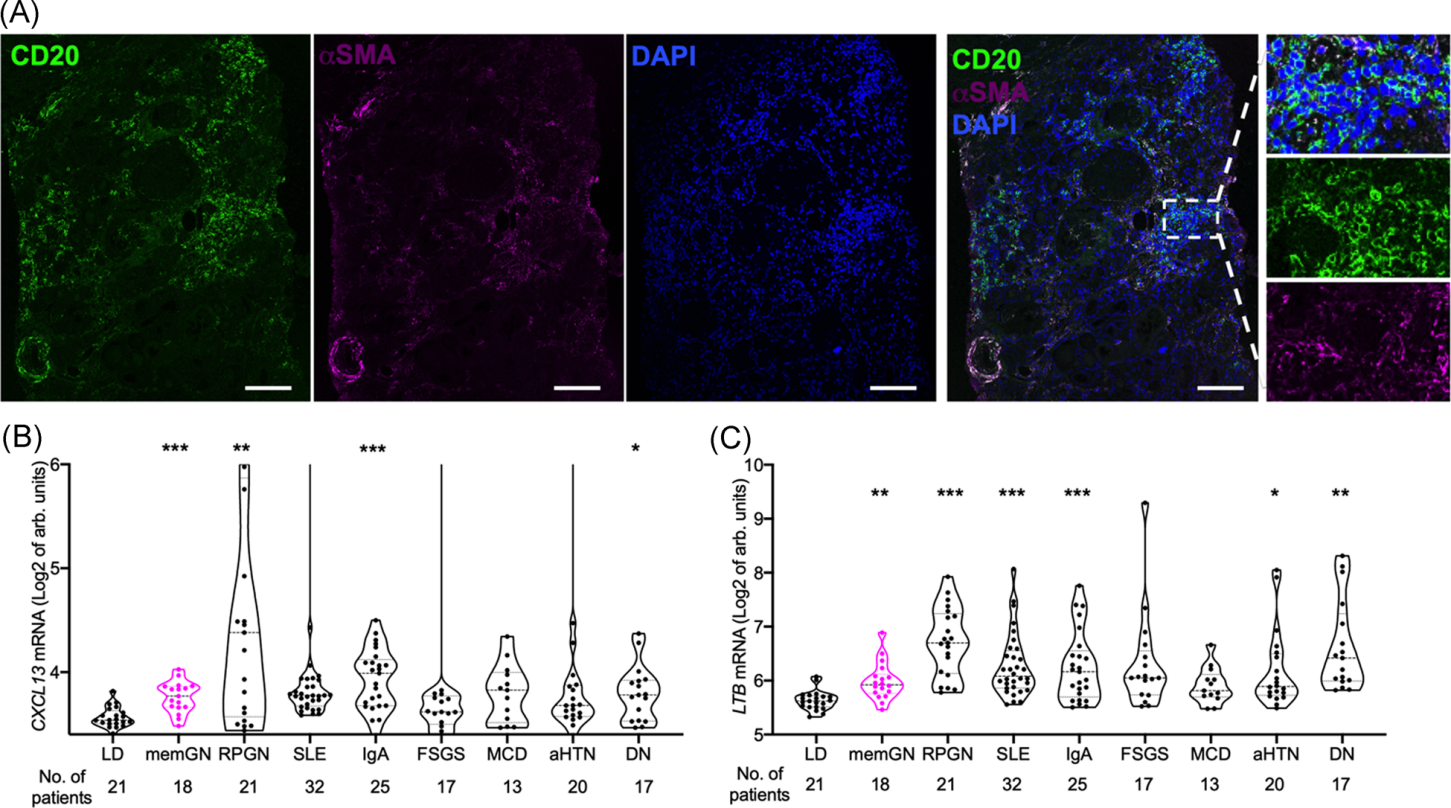
Lymphoid structures and expression of its organizers CXCL13 and LTB are detected in human membranous glomerulopathy. (A) Confocal microscopy after immunostaining for B cells (CD20, green) and stroma (α‐smooth muscle actin, purple) with nuclear counterstain (DAPI, blue) in membranous glomerulopathy (single stains, merged figure and magnified inserts of organized lymphoid structure, bar indicates 100 μm). (B) and (C) Renal interstitial *CXCL13* (B) and *LTB* (C) expression was assessed in biopsies from living kidney donors (LD), and patients with a histologic diagnosis of membranous glomerulopathy (memGN), rapid progressive glomerulonephritis (RPGN), renal systemic lupus erythematodes (SLE), IgA nephropathy (IgA), focal and segmental glomerulosclerosis (FSGS), minimal change disease (MCD), hypertensive nephropathy (aHTN), and diabetic nephropathy (DN) (Dunn's after Kruskal–Wallis test, GSE104948 and GSE104954, patient numbers for each condition)

Immunostaining for B cell marker CD20 and structural marker αSMA was assessed in human kidney biopsies with membranous nephropathy (Figure 1A). Confirming earlier findings of B cell infiltrates,^9^, ^10^ it additionally revealed organized structures, which conform to the definition of TLS.^1^

To address a broader range of renal diseases, mediators of TLS formation CXCL13^1^, ^8^ and lymphotoxin B (LTB)^7^, ^9^ in renal interstitium were analyzed in gene expression data sets from 195 human biopsies (Figure 1B,C, ^26^). Expectedly, both increased in highly inflammatory conditions such as crescentic glomerulonephritis, renal systemic lupus erythematodes, and IgA nephropathy compared with healthy kidneys from living organ donors. In addition, there was also a significant rise in more chronic conditions including in diabetic nephropathy and membranous glomerulopathy.

These measurements are consistent with the presence of tertiary lymphoid structures in human kidney disease, namely membranous glomerulopathy.

### 3.2 B cell infiltrates in membranous glomerulopathy associate with decreased excretory kidney function

**Figure 2:**
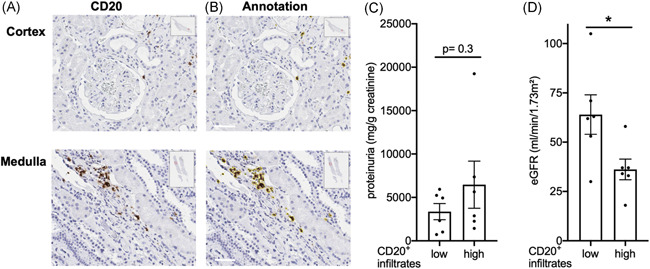
B cell infiltrates associate with decreased excretory kidney function, but not proteinuria in membranous glomerulonephritis. (A)–(D) Renal biopsies with a diagnosis of membranous glomerulonephritis were assessed for CD20^+^ B cell density by digitally assisted pathology. (A) and (B) Examples of whole slide images (inserts) with stained cortical and medullary areas (A) and the results of annotation in yellow (B, bars indicate 50 μm). (C) and (D) Proteinuria (C) and eGFR (D) in patients with renal B cell infiltration above and below median at start of therapy (*t*‐tests with Welch's correction)

To investigate the functional relevance of B cell infiltrates in membranous nephropathy, we assessed their extent by digitally assisted image analysis (Figure 2A,B). Patients were divided according to B cell infiltrate abundance at start of B cell depletion therapy. Proteinuria, a major manifestation of membranous glomerulopathy, did not differ significantly between the groups (Figure 2C), nor did membranous glomerulopathy stage (2.1 ± 0.8 vs. 2.5 ± 0.7, *p*= .3, Mann–Whitney test). However, more abundant B cell infiltrates in the kidney tissue associated with significantly worse excretory kidney function (Figure 2D).

This suggests the hypothesis that B cell infiltrates may be functionally relevant beyond induction of proteinuria.

### 3.3 Effect of long‐term B cell depletion in membranous glomerulopathy

**Figure 3:**
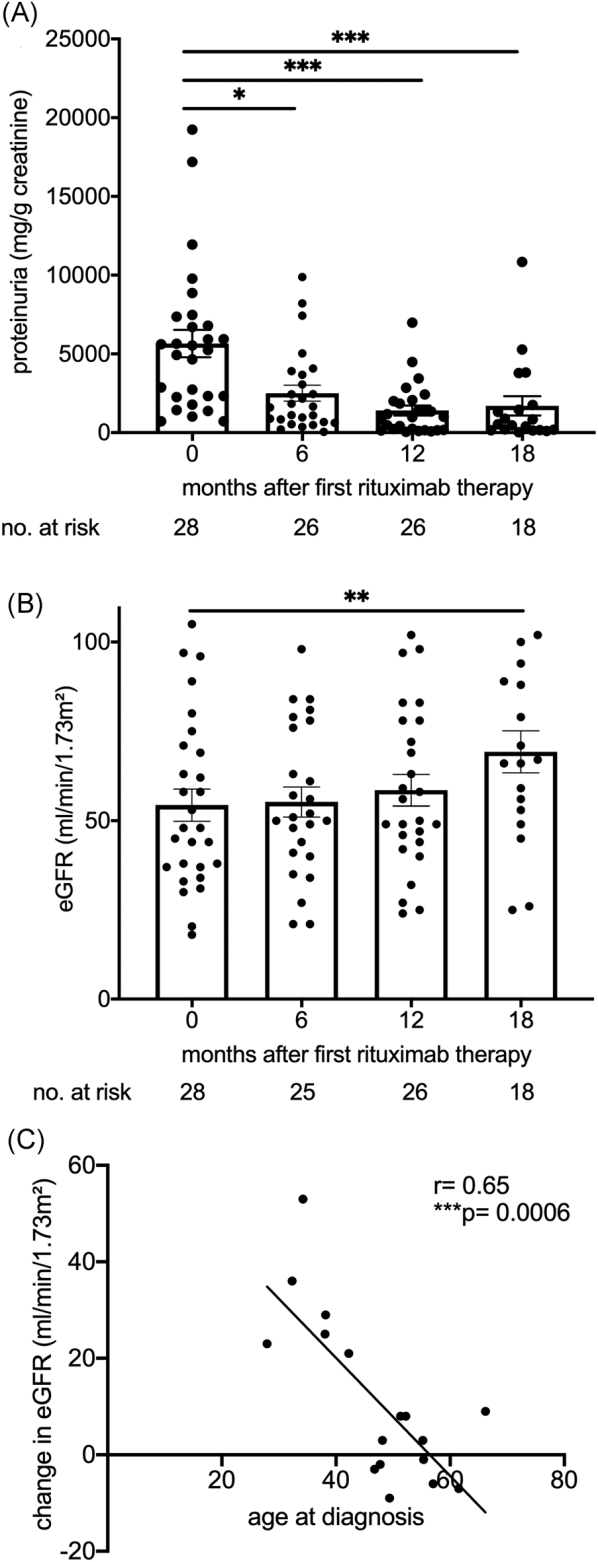
Renal function in patients with membranous glomerulonephritis treated with long‐term B cell depletion. (A)–(C) Patients with membranous glomerulopathy who responded to rituximab with a reduction in proteinuria by at least 25% at 12 months were studied. (A) and (B) Proteinuria (C, mg/g creatinine) and eGFR (B, CKD‐EPI) responses were examined over time (Dunnett's after mixed effect analysis). (C) Age at rituximab therapy in relation to change in excretory renal function expressed as eGFR after 18 months of continuous B cell depletion (Pearson's *r*). CKD‐EPI, Chronic Kidney Disease Epidemiology Collaboration; eGFR, estimated glomerular filtration rate

We next studied functional consequences of long‐term B cell depletion in this membranous nephropathy cohort. At our tertiary care institution, 35 patients with membranous glomerulopathy were treated with B cell depletion, had a follow‐up of at least 12 months and an initial eGFR above 15 ml/min/1.73 m^2^ and were included into this analysis. Similar to previous reports, 80% reduced proteinuria by more than 25% within 12 months after the first rituximab administration and were classified as responders according to the MENTOR trial definition^21^ (Table 1). The decrease of proteinuria in responders was detectable after 6 months of B cell depletion (Figure 3A).

**Table 1:** Characterization of the patients with membranous glomerulopathy

	All rituximab treated patients (*n *= 35)	Responders (*n *= 28)
Age at diagnosis (years)	47.8 ± 2.2	49.8 ± 2.3
Sex (% male)	85.7 (30)	82.1 (23)
Art. hypertension	94.3 (33)	92.9 (26)
Diabetes	17.1 (6)	21.4 (6)
PLA2R positivity	85.7 (30)	89.3 (25)
Disease characteristics at start of rituximab therapy		
Age (years)	54.2 ± 2.3	56.6 ± 2.4
Proteinuria (g/g creatinine)	5.5 ± 0.7	5.8 ± 8.9
Serum creatinine (μmol/L)	140 ± 9.4	138 ± 9.3
eGFR (CKD‐EPI, ml/min/1.73 m^2^)	56.0 ± 4.5	54.3 ± 4.5
No. of antihypertensive medications	1.92 ± 0.16	2.05 ± 0.19
Disease duration since diagnosis (months)	71.5 ± 13.0	77.2 ± 15.9
No. of previous immunosuppressive therapies	1.24 ± 0.17	1.15 ± 0.20
Previous calcineurin inhibition	65.7 (23)	60.7 (17)
Rituximab therapy		
Number of infusions (month 18)	3.1 ± 0.3	2.9 ± 0.2
Cumulative dose until month 18 (g)	2.6 ± 0.3	2.5 ± 0.2
Other immunosuppression at month 18		
Steroids		28% (5)
Other agents		11% (1)

*Note*: Data are expressed as % (*n*) and mean ± *SEM* unless stated otherwise.

Abbreviations: CKD‐EPI, Chronic Kidney Disease Epidemiology Collaboration; eGFR, estimated glomerular filtration rate; PLA2R, phospholipase 2 receptor positivity (serum and/or renal histology).

If B cell accumulation is pathophysiologically relevant, long‐term depletion should improve eGFR beyond effects on proteinuria. Indeed, kidney function quantified by eGFR improved, but reached significance only after 18 months (Figure 3B). We further addressed clinical factors that may affect renal functional recovery. Calcineurin inhibitor withdrawal can improve eGFR and these drugs were prescribed to 54% of our cohort at various times before rituximab administration. However, this therapy did not significantly associate with recovery of excretory renal function (Table 2). The marked delay in eGFR improvement also argues against hemodynamic effects.

**Table 2:** Univariate correlation of change in proteinuria (PU) and in renal excretory function (eGFR) after 18 months of B cell depletion in the membranous glomerulopathy patient cohort

	Total cohort		Responder	
	PU	eGFR	PU	eGFR
Sex (male = 1)	−0.11 (0.61)	−0.09 (0.67)	0.03 (0.89)	−0.14 (0.59)
PLA2R status at diagnosis	−0.33 (0.13)	0.17 (0.43)	−0.26 (0.30)	0.12 (0.64)
Age at diagnosis	0.09 (0.69)	−0.37 (0.08)	0.37 (0.13)	**−0.66 (0.0027)
Age at start of rituximab	0.12 (0.58)	−0.39 (0.07)	0.40 (0.1)	***−0.81 (<0.0001)
Time from diagnosis to rituximab	0.10 (0.64)	−0.13 (0.56)	0.10 (0.69)	−0.40 (0.10)
Previous immunosuppressive therapy	0.11 (0.61)	−0.36 (0.09)	0.06 (0.83)	**−0.60 (0.008)
Previous calcineurin inhibition	0.24 (0.25)	−0.06 (0.80)	0.25 (0.31)	−0.25 (0.31)
eGFR at start of rituximab	−0.17 (0.44)	*−0.498 (0.02)	−0.08 (0.76)	−0.32 (0.19)
Proteinuria at start of rituximab	−0.20 (0.34)	0.1554 (0.49)	0.08 (0.76)	0.14 (0.60)
Relative proteinuria at 18 months	N.a.	−0.26 (0.26)	N.a.	−0.31 (0.25)
Reduction in antihypertensive drug no.	**0.53 (0.009)	*−0.44 (0.04)	0.44 (0.08)	−0.41 (0.11)

*Note*: Correlations are given as Pearson's for parametric and Spearman's *r* for nonparametric values (*p* value). Significant *p* values are indicated by asterisk.

Abbreviations: eGFR, estimated glomerular filtration rate; N.a., not applicable; PLA2R, phospholipase A 2 receptor.

Previous immunosuppression and age, both at diagnosis and at first B cell depletion, negatively associated with renal recovery. Improvement in eGFR significantly inversely correlated with age at diagnosis (Figure 3C). Age and previous therapies, but not the time from diagnosis to B cell depletion, remained a significant factor in multivariable linear regression analysis (Table 3).

**Table 3:** Multivariable correlation of estimated glomerular filtration rate (eGFR) change with clinical characteristics after 18 months of B cell depletion in responding patients (*r* ^2^ = 0.62, adjusted *r* ^2^ = 0.55)

Variable	Estimate	Standard error	*p* value
Age at rituximab	−0.80798	0.22023	.0021**
Previous immunosuppressive therapy	−6.73755	2.84448	.0308*
Time from diagnosis to rituximab	0.02492	0.03987	.5408

* *p* < .05

** *p* < .01.

These results demonstrate a functional improvement with long‐term B cell depletion for membranous glomerulopathy in humans.

### 3.4 Improvement of kidney function with long‐term B cell depletion in CKD of unknown origin

**Figure 4:**
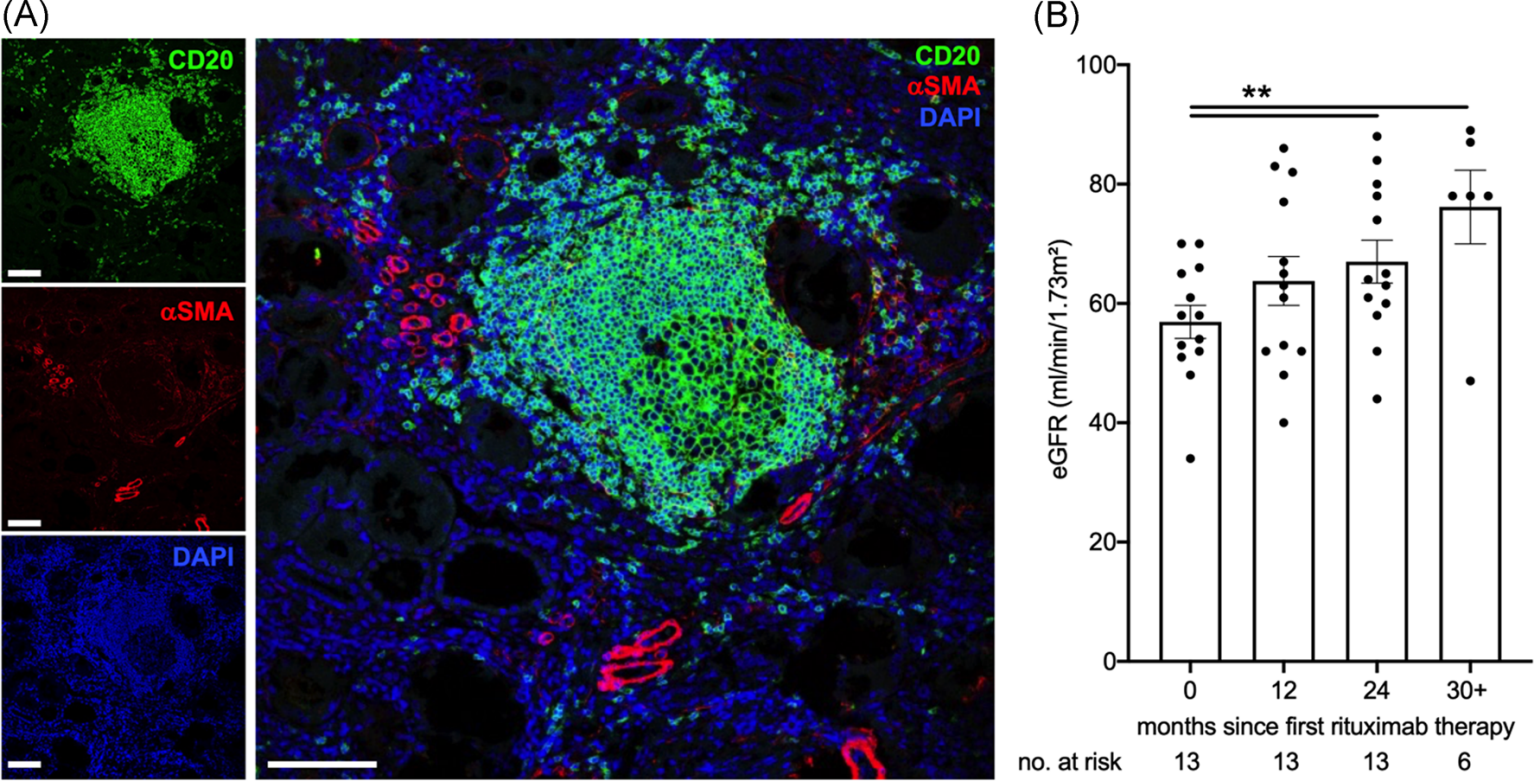
Marked tertiary lymphatic structure in the absence of autoimmunity and improved excretory kidney function in patients with renal impairment receiving long‐term B cell depletion for neurological conditions. (A) A nephrectomy specimen from a patient with terminal kidney failure due to oxalosis was stained for B cells (CD20, green) and stroma (α‐smooth muscle actin, red) with nuclear counterstain (DAPI, blue) (single stains and merged figure, bars indicate 100 μm). (B) Renal function was assessed in neurological patients with CKD (eGFR 70 ml/min/1.73 m^2^ or less) who received B cell depletion for 24 months or longer (*n *= 13, Dunnett's after mixed effect analysis). CKD, Chronic kidney disease; eGFR, estimated glomerular filtration rate

B cell infiltrates have been found in a variety of kidney conditions beyond membranous nephropathy. As an example of nonprimarily antibody‐mediated kidney disease, we detected a large tertiary lymphoid organ including a germinal center in a nephrectomy specimen from a patient with oxalosis (Figure 4A). If functionally relevant, their removal should be of benefit beyond well‐defined autoimmune disease.

To start to address this hypothesis, we studied eGFR in patients receiving chronic B cell depletion for neurological conditions with an eGFR of 70 ml/min/1.73 m^2^ or less. As detailed in methods, patients with a formal diagnosis of renal disease, namely glomerulonephritis, were excluded. Clinical and laboratory characteristics are shown in Table 4. eGFR significantly increased after two years of therapy (Figure 4B). The rise is very probably even underestimated due to improved neurological function and muscle mass.

**Table 4:** Characterization of the neurological patients (eGFR < 70 ml/min/1.73 m^2^, *n* = 13)

Age at start of rtx therapy (years)	58.1 ± 3.6
Sex (% male)	15% (2)
Underlying neurological disease	
Autoimmune encephalitis	3
Neuromyelitis optica spectrum disorders (NMOSD)	2
Cerebral vasculitis	5
Multiple sclerosis	2
Autoimmune neuromuscular diseases	1
Clinical characterization	
Art. hypertension	62% (8)
Diabetes	15% (2)
Serum creatinine	103 ± 7 μmol/L
eGFR (CKD‐EPI, ml/min/1.73 m^2^)	57 ± 3 ml/min
Dipstick proteinuria	42% (6)
Dipstick hematuria	33% (4)
Immunosuppression	
Number of rituximab infusions (month 24)	4.7±0.2
Cumulative rituximab dose until month 24 (g)	6.1±0.4
Previous immunosuppression	
Steroids	100% (13)
Other agents	100% (13)
Immunosuppression at month 24	
Steroids	67% (8)
Other agents	33% (4)

*Note*: Data are expressed as % (*n*) and mean ± *SEM* unless stated otherwise. Urine dipstick analysis was performed in 12/13 patients.

Abbreviations: CKD‐EPI, Chronic Kidney Disease Epidemiology Collaboration; eGFR, estimated glomerular filtration rate.

These data propose that chronic B cell depletion could confer a beneficial effect in CKD independent of a specific pathologic entity.

## 4 DISCUSSION

Our data demonstrate improvement in kidney function with long‐term B cell depletion. Given that organized B cell infiltrates develop in a range of renal diseases of various etiologies, these results encourage their further investigation as therapeutic targets beyond autoimmune disease.

In membranous glomerulopathy, we observed improvement of excretory kidney function after 18 months of B cell depletion. This agrees with other reports.^28^, ^29^, ^30^, ^31^ No eGFR improvement was reported in the rituximab‐treated participants of the MENTOR trial.^21^ However, these patients received their last course B cell depletion in month 6 of the study. Similar considerations apply to the GEMRITUX study, which reported a stable excretory renal function after 6 months^22^ and the single rituximab dose given in the STARMEN study.^32^ This underlines the need for extended observations of therapeutic success in membranous nephropathy after B cell depletion. Our data from patients with CKD of unknown origin propose that this may also apply to other forms of kidney disease, as suggested by murine experimental models.^16^ It agrees with significant upregulation of TLS gene expression also in human RPGN, IgA nephropathy, and diabetic nephropathy found in our analysis. A better understanding of mechanisms of TLS formation, including interaction with blood vessels and organ‐specific cues^1^, ^9^, ^16^ is needed to extend therapeutic approaches beyond B cell depletion.

Therapeutic regimens in membranous nephropathy have developed rapidly in recent years.^20^, ^33^ The proteinuria response rate in our tertiary care cohort corresponds to similar reports^28^, ^29^, ^34^ and was higher than in others, who received lower doses of rituximab.^30^, ^35^ In agreement with previous studies, no single clinical characteristic associated with proteinuria response to rituximab. Importantly, similar to most^36^ but not all reports,^35^ a lower eGFR did not adversely affect proteinuria response, nor did advanced age.^37^ Our present analysis is limited by incomplete PLA2R measurements, which have been associated with proteinuria response in membranous glomerulonephritis.^22^, ^35^, ^38^ Concomitant medication including corticosteroids as are also administered with rituximab infusions may have influenced response. Calcineurin inhibitor withdrawal may acutely improve renal function, as reported for membranous nephropathy in the MENTOR trial and others.^21^, ^31^, ^39^ Calcineurin inhibitors are part of combined immunosuppressive approaches also in severe membranous nephropathy.^31^, ^32^ They are known to promote kidney fibrosis.^40^, ^41^ It is reassuring that they did not preclude long‐term renal recovery in patients of our cohort. Collectively, our observations further strengthen the evidence of effective proteinuria reduction and a low rate of adverse events during B cell depletion for primary membranous glomerulopathy.

Aging adversely affected renal regeneration in a number of experimental and clinical studies.^42^, ^43^, ^44^ Biologically aged kidneys are challenged to provide enough cell divisions for functional regeneration. In addition, enhanced glomerular aging has been described in glomerular disease including membranous glomerulopathy.^42^, ^45^ While we are not aware that a negative correlation of age and regeneration has been reported previously in membranous glomerulopathy, our data agree with results of patients with the nephrotic syndrome due to other etiologies including minimal change disease and focal and segmental glomerulosclerosis.^46^ Here, an improved eGFR was observed after 1 year of B cell depletion exclusively in younger patients, mainly children. Our data argue for an age‐dependent factor in regeneration after successful therapy also in membranous glomerulopathy. Our responder cohort comprised patients aged 28–73 years at diagnosis and 33–77 years at first rituximab therapy, with a lag time between diagnosis and B cell depletion of 0–318 months (mean 77 months). These values are expectedly strongly correlated. The large age range enabled us to start to explore whether a delay in therapy contributed to impaired eGFR improvement. Our data do not indicate a deleterious effect of delayed rituximab therapy.

Our study is also limited by the fact that there were too few patients with biopsy material for B cell staining and long‐term follow‐up for quantitative association with the extent of eGFR response (*n *= 7). We employed computer‐assisted quantification of the whole slide to obtain maximal information on B cell abundance in the kidney. However, given their scattered nature, even a complete biopsy core may not reflect the total extent. Advances in imaging and possibly antigen‐specific contrast media could provide novel approaches to quantify and longitudinally monitor B cell infiltrates in renal disease in the future.

In summary, our data propose an additional rationale for the use of long‐term B cell depletion in kidney diseases by disruption of local B cell‐dominated structures. Extended controlled trials including serial histologic assessments are necessary to validate this approach. Our results may encourage systematic analysis of renal functional development in additional cohorts.

## AUTHOR CONTRIBUTIONS

Susanne V. Fleig, Christoph Schröder, Hermann Haller, Thomas Skripuletz, and Sibylle von Vietinghoff designed research. Susanne V. Fleig, Franz Felix Konen, Christoph Schröder, Jessica Schmitz, Stefan Gingele, Jan Hinrich Bräsen, and Svjetlana Lovric acquired data. Franz Felix Konen, Christoph Schröder, BMWS, and Sibylle von Vietinghoff analyzed data. Susanne V. Fleig, Franz Felix Konen, Christoph Schröder, and Sibylle von Vietinghoff wrote the manuscript with help from all coauthors, all authors read and approved the manuscript.
